# An Anthocyanin-Based Visual Reporter System for Genetic Transformation and Genome Editing in Cassava

**DOI:** 10.3390/ijms252111808

**Published:** 2024-11-03

**Authors:** Xing-Hou Zhen, Ran-Ran Pan, Xiao-Hua Lu, Yu-Jian Ge, Rui-Mei Li, Jiao Liu, Ya-Jie Wang, Ke-Xian Yi, Chun-Xia Li, Jian-Chun Guo, Yuan Yao, Meng-Ting Geng

**Affiliations:** 1 National Key Laboratory for Tropical Crop Breeding, School of Life and Health Sciences, Hainan University, Haikou 570228, China; 21110710000044@hainanu.edu.cn (X.-H.Z.); 19071010110006@hainanu.edu.cn (X.-H.L.); chun_xia_li@126.com (C.-X.L.); guojianchun@itbb.org.cn (J.-C.G.); 2 National Key Laboratory for Tropical Crop Breeding, Key Laboratory of Biology and Genetic Resources of Tropical Crops, Sanya Research Institute, Institute of Tropical Bioscience and Biotechnology, Chinese Academy of Tropical Agricultural Sciences, Haikou 571101, China; 18789085180@163.com (R.-R.P.); liruimei@itbb.org.cn (R.-M.L.); liujiao@itbb.org.cn (J.L.); wangyajie@itbb.org.cn (Y.-J.W.); 3 School of Tropical Agriculture and Forestry, Hainan University, Haikou 570228, China; 22220951310056@hainanu.edu.cn; 4 Environment and Plant Protection Institute, Sanya Research Institute, Chinese Academy of Tropical Agricultural Sciences, Haikou 571101, China; yikexian@126.com

**Keywords:** cassava anthocyanins, genetic transformation, genome editing, visual reporter

## Abstract

Cassava (*Manihot esculenta* Crantz) is a staple crop in tropical and subtropical regions, valued for its high starch content in roots. Effective genetic transformation and genome editing of cassava require efficient screening methods for transgenic and edited plants. In this study, a visual selection marker system using an R2R3-MYB transcription factor anthocyanin 1 gene (*HbAN1,* LOC110667474) from a rubber tree (*Hevea brasiliensis* Müll. *Arg*.) has been developed to facilitate the identification of transgenic cassava plants. Transgenic cassava lines expressing *HbAN1* accumulated anthocyanins in their leaves, allowing for easy visual identification without the need for destructive assays or specialized equipment. Importantly, the accumulation of anthocyanins did not affect the regeneration or transformation efficiency of cassava. Additionally, the AR-CRISPR/Cas9-gRNA system with the *HbAN1* gene as a marker produced *MeCDD4* gene-edited cassava mutants with purple leaves, demonstrating successful editing. This anthocyanin-based visual reporter (AR) system will provide an effective tool for genetic transformation and genome editing in cassava.

## 1. Introduction

Cassava (*Manihot esculenta* Crantz) is a tropical staple crop for people in tropical and subtropical regions [1]. It has a high yield and is tolerant to drought and barren land. The tuberous roots of cassava have a high starch content (about 80% of the dry matter) [2]. Cassava starch is an important source of calories, providing the staple food of 800 million people worldwide [3]. Meanwhile, it is suitable for non-food industrial utilization uses, including ethanol production, paper and textile production, and chips [4]. Genetic transformation and gene editing are important means to improve cassava’s biotic stress, abiotic stress, yield, and starch quality. In 1996, two research groups reported breakthrough results of cassava gene transformation in the model cultivar 60444 [5,6]. After several years of development, cassava genetic transformation technology has gradually matured, and protocols for some local planting cultivars in Africa, South America, and Asia have been established, such as TME204 [7], T200 [8], TME14 [9], Verdinha [10] and SC8 [11]. In 2017, the first report of CRISPR/Cas9 technology employed to knockout the phytoene desaturase gene (*MePDS*) in cassava was published [12]. Several genes were knocked out to enhance cassava’s resistance to disease [13], increase amylose content [14], and obtain amylose-free cassava [15]. Great progress has been made in the genetic transformation of cassava. However, the molecular identification of transgenic seedlings is cumbersome and time-consuming, necessitating the establishment of efficient transgenic monitoring methods.

The development of visual reporter systems allows for efficient assessment of the T-DNA transfer into the genome and selection of T-DNA-free edited lines in the self-crossed progeny. Visual reporter genes have been used for cassava transformation, including β-glucuronidase (GUS) [16], firefly luciferase (LUC) [17], green fluorescent protein (GFP) [7], and red fluorescent protein (DsRed) [18]. These visual reporter genes have been widely used in plant genetic transformation [19]. The limitations of GUS and LUC as transgenic selection markers are that GUS requires destructive sampling, while LUC is substrate-dependent and demands specialized equipment for detection. Additionally, GUS can yield false positives due to background enzymatic activity, and LUC’s fluorescence signal decays over time, complicating long-term monitoring. GFP and DsRed require specialized equipment for fluorescence excitation and detection, which add complexity and cost to the screening process. Additionally, natural autofluorescence in cassava cells can interfere with these markers, reducing the clarity and accuracy of detection. Several reports have demonstrated the potential of anthocyanins as visual reporters, as anthocyanin accumulation can be easily observed with the naked eye [20]. Anthocyanins are a class of water-soluble natural pigments synthesized in plants, typically combined with one or more monosaccharides or polysaccharides to form anthocyanidins [21]. Anthocyanins are considered a safe, non-toxic visual marker that could reduce concerns about the safety of transgenic plants [22].

Overexpression of key regulatory transcription factors in the anthocyanin synthesis pathway can promote the accumulation of anthocyanins in plants. PtrMYB119 from *Populus trichocarpa* positively regulates anthocyanin production in poplar and has no adverse effects on plant growth [23]. The bHLH transcription factor ThMYC4E from common wheat (*Triticum aestivum*) activated anthocyanin biosynthesis in the transgenic lines [24]. Overexpression of the *MdMYB24L* gene from apple (*Malus pumila* Mill.) increased anthocyanin contents in the transgenic apple calli [25]. Transcription factor FvTCP9 promotes anthocyanin biosynthesis in the transient fruits of strawberry (*Fragaria vesca*) [26]. R2R3-MYB transcription factor anthocyanin 1 and 2 (HbAN1 and HbAN2) from *H. brasiliensis* have been proven to enhance anthocyanin accumulation in transgenic tobacco (*Nicotiana tabacum* cv. Xanthi) [27,28], and *HbAN2*-overexpressing embryos and regenerated roots of *H. brasiliensis* accumulate more anthocyanins [28]. Both *H. brasiliensis* and cassava belong to the *Euphorbiaceae* species. Therefore, we hypothesized that HbAN1 and HbAN2 could promote anthocyanin accumulation in cassava.

In this study, *HbAN1* was transformed and expressed in cassava to investigate whether it could promote anthocyanin accumulation in cassava, aiming to potentially establish a visual reporter system for genetic transformation and genome editing in this crop.

## 2. Results

### 2.1. Acquisition of HbAN1-Overexpressing Transgenic Cassava

**Figure 1:**
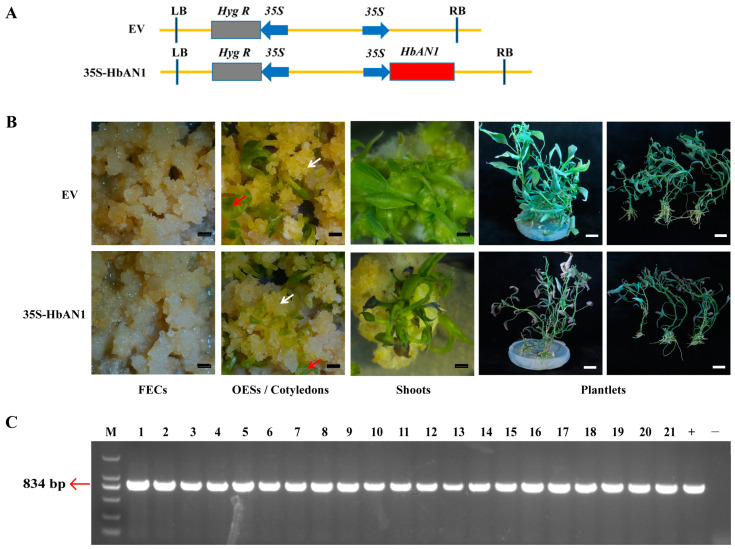
Cassava cultivar SC8 genetic transformation of *HbAN1* overexpression vector. (**A**) Vector schematics of EV and 35S-HbAN1. RB and LB symbolize the right and left borders of T-DNA, respectively. (**B**) Genetic transformation processes of EV and 35S-HbAN1 in SC8. FECs represent friable embryogenic calli, and OESs represent somatic organized embryogenic structures. White arrows represent OESs, and red arrows represent cotyledons. Black scale bars represent 5 mm, and white scale bars represent 20 mm. (**C**) Molecular characterization of the regeneration plantlets with purple leaf tips. M, DL2000 DNA Maker; 1–21, serial number of regeneration plantlets; +, Positive control; -, Negative control.

To investigate whether HbAN1 activates anthocyanin accumulation in cassava, *HbAN1* under the control of 35S promoter (vector name: 35S-HbAN1) and the empty vector as the control (vector name: EV) were genetically transformed into friable embryogenic calli (FECs) of cassava cultivar SC8 through Agrobacterium LBA4404 (Figure 1A). A phenotypic evaluation revealed that at the stage of FECs, somatic organized embryogenic structures (OESs), and cotyledon regeneration, the color of these tissues was similar between EV and 35S-HbAN1 lines (Figure 1B). However, during the period of plantlet regeneration, the shoots and plantlets transformed with 35S-HbAN1 appeared more purple at the leaf tips compared with the EV (Figure 1B). The regeneration plantlets with purple leaf tips were performed by molecular characterization; the results showed that all of them contained the *HbAN1* gene, so they were positive lines (Figure 1C).

### 2.2. Evaluation of HbAN1-Overexpressing Genetic Transformation

**Figure 2:**
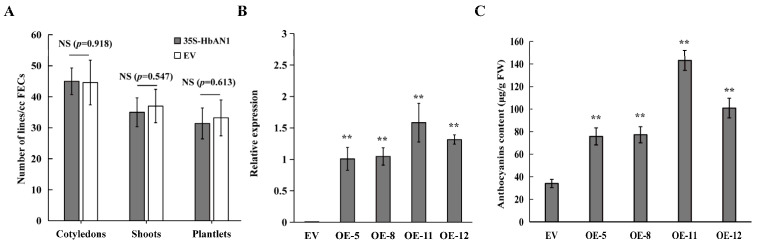
Evaluation of *HbAN1*-overexpressing genetic transformation and anthocyanin content measurements in leaves of transgenic lines. (**A**) Numbers of cotyledons, shoots, and plantlets of EV and 35S-HbAN1 transgenic plants. (**B**) The *HbAN1* relative expression levels in leaves of EV and 35S-HbAN1 transgenic plants. (**C**) Anthocyanin contents in leaves of EV and 35S-HbAN1 transgenic plants. The data were shown as means and SD of three replicates. NS, non-significant differences in comparison to EV at *p* > 0.05 (Student’s *t*-test). **, significant differences in comparison to EV at *p* < 0.01 (Student’s *t*-test).

It has been reported that the accumulation of anthocyanins may affect plant regeneration [22]. Our results showed that the numbers of cotyledons, shoots, and plantlets between EV and 35S-HbAN1 were non-different (Figure 2A). The expressions of *HbAN1* in transgenic lines were assessed by qRT-PCR, indicating that *HbAN1* was successfully expressed in the cassava cultivar SC8 (Figure 2B). The anthocyanin contents in leaves of transgenic lines were measured by the Plant Anthocyanin Content Assay Kit (Comin, Suzhou China). The results showed that total anthocyanin contents were significantly higher in *HbAN1*-transformed plants than in EV-transformed plants (Figure 2C). These results indicate that *HbAN1* promotes cassava cultivar SC8 anthocyanin accumulation in leaves and can be used as a visual reporter gene for cassava genetic transformation.

### 2.3. Construction of AR-CRISPR/Cas9-gRNA Editing Vector and Genetic Transformation

**Figure 3:**
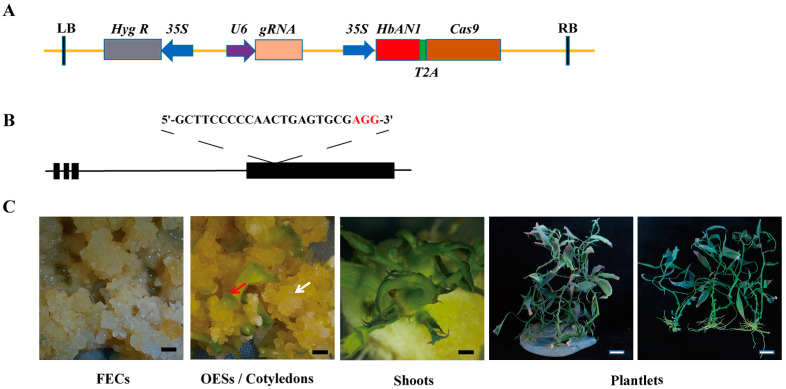
Cassava cultivar SC8 genetic transformation of AR-CRISPR/Cas9-*MeCDD4* gRNA vector. (**A**) AR-CRISPR/Cas9-*MeCDD4* gRNA vector schematic. (**B**) sgRNA designed for *MeCDD4.* Lines represent introns, and black boxes represent exons. (**C**) Genetic transformation process of AR-CRISPR/Cas9-*MeCDD4* gRNA vector in SC8. FECs represent friable embryogenic calli, and OESs represent somatic organized embryogenic structures. White arrows represent OESs, and red arrows represent cotyledons. Black scale bars represent 5 mm, and white scale bars represent 20 mm.

The gene editing vector of AR-CRISPR/Cas9-*MeCDD4*-gRNA constructed in this study was based on the CRISPR/Cas9 binary vector previously reported [29]. The full length of *HbAN1* (primers were listed in Table S1) was amplified without stop codon and inserted in front of *Cas9* to form a single open reading frame, in which *HbAN1* and *Cas9* were linked by sequences that encode 2A peptides (Figure 3A). Upon transcription, 2A peptides enable the encoding of two different genes, which are linked in a single transcript, to produce two independent proteins [30]. To test the functionality of the AR-CRISPR/Cas9- gRNA system in cassava, a guide RNA targeted against the fourth exon *MeCDD4* gene of cassava was designed (Figure 3B). During the genetic transformation of cassava using the AR-CRISPR/Cas9-*MeCDD4* gRNA vector, anthocyanin accumulation occurred in shoots and leaves (Figure 3C).

### 2.4. Analysis of the Editing Effects of the MeCDD4 Gene

**Figure 4:**
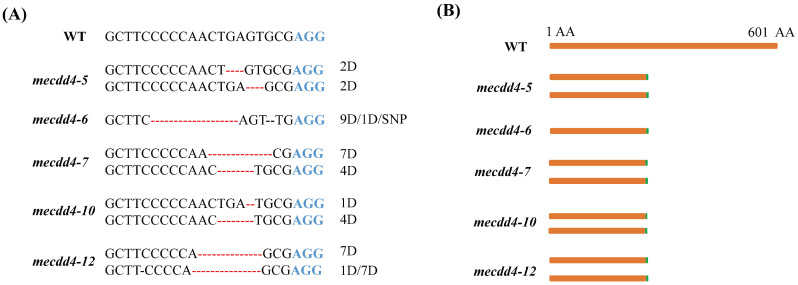
MeCDD4 gene-editing forms. (**A**) Editing forms of *mecdd4-5*, *mecdd4-6, mecdd4-7, mecdd4-10*, and *mecdd4-12.* Red dashed lines represent bases deletion, D represents deletion, and SNP represents single nucleotide polymorphism. (**B**) Frame shift mutation forms of *mecdd4-5*, *mecdd4-6, mecdd4-7, mecdd4-10*, and *mecdd4-12*.

A total of 12 regenerated plantlets with purple leaf tips were obtained and subjected to Hi-TOM sequencing to identify the editing effects of the *MeCDD4* gene. The results revealed that *MeCDD4* genes were edited in 10 of the 12 lines, resulting in an editing efficiency of 83.33% (Table 1). The editing forms of the *MeCDD4* gene were all characterized by base deletions. Lines of *mecdd4-5*, *mecdd4-6, mecdd4-7, mecdd4-10*, and *mecdd4-12* exhibited frame shift mutations (Figure 4A,B). The results indicate that the AR-CRISPR/Cas9-gRNA system can be utilized for visual screening of transgenic cassava seedlings using anthocyanin as a marker, and it enables efficient screening of genome editing cassava.

## 3. Discussion

This study demonstrates that the expression of the *HbAN1* gene in cassava cultivar SC8 could promote the accumulation of anthocyanins in cassava leaves. Anthocyanin synthesis in plants is primarily regulated by the MBW (MYB-bHLH-WD40) complex, which is composed of three types of transcription factors: R2R3-MYB, bHLH, and WD40 transcription factors [31]. Among these, R2R3-MYB TFs are key factors in regulating anthocyanin synthesis [32]. HbAN1 is an R2R3-MYB transcription factor associated with anthocyanin synthesis in rubber trees, mainly expressed in young, bronze-phase leaves [27]. When *HbAN1* was heterologously expressed in tobacco, it led to significant anthocyanins accumulation in various tissues, including leaves, petals, receptacles, and filaments [27]. Our study found that during the genetic transformation of cassava cultivar SC8 with *HbAN1*, the transgenic somatic embryos did not exhibit purple anthocyanins accumulation, while transgenic cassava leaf tips accumulated anthocyanins (Figure 1B). HbAN1 may activate the expressions of anthocyanin biosynthesis genes in transgenic cassava leaves.

Although anthocyanins are effective as a visual selection marker, excessive anthocyanins in transgenic plants may have detrimental effects on regeneration efficiency and growth of plants [22]. Specifically, high-level anthocyanins in regenerated apple and strawberry shoots were observed to cause cell toxicity, potentially due to disruptions in cellular redox balance or nutrient sequestration, leading to plant death in some cases [22]. HbAN2 can serve as a visual marker gene for the genetic transformation of rubber trees, allowing for rapid screening of the transgenic somatic embryos through anthocyanin coloration [28]. However, the accumulation of anthocyanins inhibited the development of transgenic rubber tree somatic embryos into seedlings and prevented the acquisition of transgenic plants [28]. In this study, our results found that the expression of *HbAN1* did not promote the visible accumulation of anthocyanins in cassava cultivar SC8 somatic embryos but rather enhanced the accumulation of anthocyanins in transgenic cassava leaf tips, which ultimately did not affect the transformation efficiency or regeneration efficiency of cassava cultivar SC8 (Figure 1B and Figure 2A).

Genome editing technology is a crucial tool for improving cassava. However, the presence of CRISPR/Cas9 components in edited cassava may raise safety concerns regarding its application. Eliminating these components through selfing ensures that the edited plants are free of foreign DNA, thereby enhancing their safety and suitability for practical uses [33]. A gene-editing technique by incorporating a 35S-GFP expression cassette into the editing vector has been developed. This method uses GFP fluorescence as a marker and enables the efficient selection of transgene-free plants in later generations [7]. Liu et al. (2019) incorporated the Arabidopsis *AtPAP1* gene, which regulates anthocyanins accumulation, and the tobacco *NtFT* gene, which controls flowering, into a CRISPR/Cas9 gene editing vector [34]. This created an early flowering and visual selection marker system that could accelerate the generation and identify the target gene-edited and transgene-free plants within a single, short generation [34]. In this study, a visual selection marker system designed as AR-CRISPR/Cas9-gRNA using *HbAN1* has been developed for cassava gene editing. This system successfully produced *MeCDD4* gene-edited cassava mutants with purple leaves, providing a potential method for efficient selection of gene-edited cassava plants without transgenic components in subsequent generations.

This study has demonstrated that *HbAN1* effectively promotes anthocyanin accumulation in cassava leaves, leading to the establishment of an anthocyanin-based visual reporter system for efficient screening of transgenic-positive cassava lines. However, the accumulation of anthocyanins in transgenic cassava leaves possesses antioxidant properties and could enhance stress tolerance, which could potentially interfere with results in overexpression assays that intend to validate the functions of stress-related genes. Therefore, the application of this anthocyanin-based visual reporter system should be carefully considered according to the specific objectives of each experiment.

Our research has shown that HbAN1, an R2R3-MYB transcription factor originating from the rubber tree, is capable of enhancing anthocyanin synthesis in cassava. Notably, both rubber trees and cassava are members of the *Euphorbiaceae* family. However, HbAN1 may not necessarily bind effectively to the promoters of anthocyanin-related genes in other plant species, so the system’s effectiveness in other plants remains to be further verified. In future research, we will assess the effectiveness of this anthocyanin-based visual reporter system in other economically significant tropical crops, such as sugarcane and bananas, to enhance the efficiency of transgenic screening in these species.

## 4. Materials and Methods

### 4.1. Plant Materials

The cultivar (*Manihot esculenta* Crantz, Shouth China no 8) ‘SC8’ is one of the main planted cassava varieties in China. The FECs of SC8 were induced according to the protocol reported in our previous study [11]. Cassava cultivar SC8 primary embryos were induced from axillary buds that were cultured on CIM medium (Supplementary Table S2) and transferred to fresh medium every twenty days. Somatic embryos (SEs) were produced during the period of primary embryo cultivation. FECs were produced from SEs. The matured SEs were divided into small pieces and transferred to Greshoff and Doy (GD) mediums (Supplementary Table S2) and cultured at 28 °C without light. After two weeks, FCEs were produced at the edge of SEs and transferred to GD medium. FECs were circularly cultured on GD medium and refreshed every month for a maximum of five to six months. Leaf samples were collected from two-month-old transgenic seedlings of EV (empty pCAMBIA1300-35S-GFP without GFP) and 35S-HbAN1 (pCAMBIA1300-35S-HbAN1). The experiments were three biological replicates, and each replicate was collected from the leaves of uniform transgenic cassava seedlings. Samples were frozen in liquid nitrogen immediately and stored at −80 °C for further experiments.

### 4.2. Construction of HbAN1 Expressing and AR-CRISPR/Cas9-MeCDD4-gRNA Editing Vectors

The overexpression vector constructed in this study was based on pCAMBIA1300-35S-GFP binary vector. The full length of *HbAN1* (P3 × 46_008472, primers listed in Table S1) was amplified and cloned into pCAMBIA1300-35S-GFP to replace GFP. The gene editing vector of AR-CRISPR/Cas9-*MeCDD4*-gRNA constructed in this study was based on CRISPR/Cas9 binary vector previously reported [29]. The full-length of *HbAN1* without stop codon was inserted in front of *Cas9* to form a single open reading frame, in which *HbAN1* and *Cas9* were linked by sequences that encode 2A peptides. Upon transcription, 2A peptides enable two different genes, which are linked in a single transcript, to produce two independent proteins [30]. A specific sgRNA (5′-GCTTCCCCCAACTGAGTGCG-3′) was designed to recognize the fourth exon in cassava *MeCDD4* (Manes.15G183500) gDNA and built into gene editing vector.

### 4.3. Cassava Genetic Transformation

The constructed vectors were transformed into *Agrobacterium tumefaciens* LBA4404 through electroporation. The transformation system of cassava cultivar SC8 was previously reported by our laboratory, so the cassava transformation in this study was operated according to the early report [11]. *Agrobacterium* LBA4404 harboring vector was cultured in YEP liquid medium containing kanamycin (50 mg/L) and rifampicin (50 mg/L) until the OD_600_ of 0.75 was achieved. Then, the *Agrobacterium* cells were collected and resuspended with GD liquid medium containing 200 µM AS to adjust the OD_600_ of 0.65. FECs were co-cultured with Agrobacterium cell solution for 30 min at 50 rpm and 28 °C and centrifuged for 10 min at 1000 rpm. Finally, co-cultured FECs were transferred onto the nylon filter mesh upon sterilized absorbent filter paper to remove excessive liquid and then transferred onto GD medium containing 200 µM AS and cultured for 3 days without light at 22 °C.

### 4.4. Extraction and Measurement of Anthocyanins

The anthocyanin contents in leaves of transgenic lines were measured by Plant Anthocyanin Content Assay Kit (Comin, Suzhou, China). The plant sample (approximately one gram) was thoroughly ground in liquid nitrogen, then the sample powders were transferred to a tube containing 1 mL extraction solution and incubated for 20 min at 75 °C. Then, they were centrifuged at 8000× *g* for 10 min at 25 °C to precipitate the plant materials. The absorbance of the supernatant liquor and control were measured at 520 nm, and the formula 33.4 × ΔA × F ÷ W was used to roughly estimate the content of anthocyanins. ΔA = A sample − A control; F represents dilution factor; W represents sample weight; unit, μg/g.

### 4.5. Statistics of Cotyledons, Bud Regenerations and Seedlings of the Regenerated Plants

The transformed FECs were circularly cultured on MSN medium containing 250 mg/L carbenicillin and 20 mg/L hygromycin, and the medium was refreshed twice a month. During the period, the number of cotyledons was recorded. The cotyledons were transferred to shoot-inducting medium containing 100 mg/L carbenicillin and 10 mg/L hygromycin, and the medium was refreshed twice a month. During this period, the number of the regenerated buds was counted. The number of the regenerated buds/the number of the cotyledons was counted as bud regeneration rate. The mature buds were transferred to MS medium containing 10 mg/L hygromycin, then some buds could grow into seedlings. During this period, the number of regenerated seedlings was recorded. The number of the regenerated seedlings/the number of the regenerated buds was counted as seedling regeneration rate.

### 4.6. Hi-TOM Sequencing

*MeCDD4* editing analysis was performed by Hi-TOM program. The target fragment was amplified by gene-specific primers (primers were listed in Table S1), and the DNAs of the transgenic positive lines were used as templates. The second-round PCRs were amplified using the first-round RCR products as templates. Then, the second-round PCR products were mixed equally and purified. Finally, the purified products were sequenced by Novogene.

### 4.7. qRT-PCR Validation

Total RNA of samples was extracted using Plant Total RNA Isolation Kit Plus (FOREGENE, Chengdu, China), and reverse transcribed into cDNA was performed using MonScript™ RTIII Super Mix with dsDNase (Monad, Beijing, China). The qRT-PCRs were performed using MonAmp™ ChemoHS qPCR Mix (Monad, Beijing, China); the *TubuLin* was used as a reference gene. All primers of the qRT-PCRs were listed in Table S1. Three replicates per sample and the data of qRT-PCR were analyzed using the 2^−ΔΔCt^ method.

## 5. Conclusions

In this study, an anthocyanin-based visual reporter system for cassava genetic transformation and genome editing was developed using the *HbAN1* gene from the rubber tree. This visual reporter system provides an efficient tool for molecular breeding in cassava. Future studies will further assess the effectiveness of this system in screening transgenic seedlings in other crops.

## Figures and Tables

**Table 1:** Gene editing analysis of *MeCDD4* gene.

Line Name	Mutation Sequence	Reads Count	Reads Ratio	Mutation Type	Mutant Base
WT	GCTTCCCCCAACTGAGTGCGAGG	68,606	83.04%	WT	
*meccd4-1*	GCTTCCCCCAA-----------TGCGAGG	26,593	46.25%	5D	CTGAG
	GCTTCCCCCAACTG------GCGAGG	24,928	43.36%	3D	AGT
*meccd4-2*	GCTTCCCCCA--------TGCGAGG	56,300	84.10%	6D	ACTGAG
*meccd4-4*	GCTT------------------------------------------	33,361	49.97%	26D	CCCCCAACTGAGTGCGAGGTGATTGA
	GCTTCC--------------------------CGAGG	26,650	39.92%	12D	CCCAACTGAGTG
*meccd4-5*	GCTTCCCCCAACT----GTGCGAGG	42,805	51.02%	2D	GA
	GCTTCCCCCAACTGA----GCGAGG	36,498	43.50%	2D	GT
*meccd4-6*	GCTTC---------------------AGT-TGAGG	23,776	90.84%	9D, 1D, SNP	CCCCAACTG,G,C->T
*meccd4-7*	GCTTCCCCCAA---------------CGAGG	32,807	45.03%	7D	CTGAGTG
	GCTTCCCCCAAC--------TGCGAGG	30,596	41.99%	4D	TGAG
*meccd4-8*	GCTTCCCCCAACTGA----GCGAGG	33,855	52.31%	2D	GT
	GCTTCCCCCAACTG------GCGAGG	13,453	20.78%	3D	AGT
	GCTTCCCCCAACT----GTGCGAGG	3718	05.74%	2D	GA
	GCTTCCCCCAACTGAGT----GAGG	3604	05.57%	2D	GC
*meccd4-9*	GCTTCCCCCA-------------TGCGAGG	22,175	36.57%	6D	ACTGAG
	GCTTCCCCCAACTGA----GCGAGG	17,791	29.34%	2D	GT
	GCTTCCCCCAACT-------------GAGG	14,399	23.75%	6D	GAGTGC
*meccd4-10*	GCTTCCCCCAACTGA--TGCGAGG	25,004	45.79%	1D	G
	GCTTCCCCCAAC---------TGCGAGG	23,782	43.55%	4D	TGAG
*meccd4-12*	GCTTCCCCCA---------------GCGAGG	32,546	44.66%	7D	ACTGAGT
	GCTT-CCCCA----------------GCGAGG	32,364	44.41%	1D,7D	C,ACTGAGT

D, base mutation form, deletion; SNP, single nucleotide polymorphism.

## Data Availability

Data are contained within the article and Supplementary Materials.

## Supplementary Materials

The following supporting information can be downloaded at: https://www.mdpi.com/article/10.3390/ijms252111808/s1.
