# Supplementary material for: An Anthocyanin-Based Visual Reporter System for Genetic Transformation and Genome Editing in Cassava
Source: Int J Mol Sci. 2024 Nov 3;25(21):11808. doi: 10.3390/ijms252111808 (PMC11547100; doi:10.3390/ijms252111808)
Supplement: Supplementary file 1 [file ijms-25-11808-s001.zip › Supplementary Tables.pdf]

Table S1 Primers used in this study

Table S1 Primers used in this study

Primer name	Primer sequencess (5'→3')	Purpose
HbAN1-F	ATGGCCGAAGGCCCTAAAGG	For *HbAn1* gene amplification
HbAN1-R	AAACATCTCATCGATCGAGG	For *HbAn1* gene amplification
MeCDD4-HT-F	ggagtgagtacggtgtgcATCCTCCAATCCGGTCATCAG	For Hi-TOM sequencing
MeCDD4-HT-R	gagttggatgctggatggTAAGCCCCATCAAGGCAAGA	For Hi-TOM sequencing
qHbAN1-F	ATGGCCGAAGGCCCTAAAGGAGT	For qPCR
qHbAN1-R	AAGATAGTTCAACCACCTCAA	For qPCR
qTUB-F	ATGCGGTTCTTGATGTTGTTC	For qPCR
qTUB-R	TCGGTGAAGGGAATACAGAGA	For qPCR

Table S2 The media and their compositions

Table S2 The media and their compositions

Medium	Compositions (1 L)
CIM	4.4 g MS, 20 g sucrose, 0.32 mg CuSO4, 12 mg picloram, pH 5.8, 8 g Agar
GD	2.75 g GD, 20 g sucrose, 12 mg picloram, pH5.8, 8 g Agar
